# The Impact of the COVID-19 Lockdown on Coaches’ Perception of Stress and Emotion Regulation Strategies

**DOI:** 10.3389/fpsyg.2020.601743

**Published:** 2021-01-12

**Authors:** Giampaolo Santi, Alessandro Quartiroli, Sergio Costa, Selenia di Fronso, Cristina Montesano, Francesco Di Gruttola, Edoardo Giorgio Ciofi, Luana Morgilli, Maurizio Bertollo

**Affiliations:** ^1^Department for Life Quality Studies, Alma Mater Studiorum – University of Bologna, Bologna, Italy; ^2^Department of Psychology, University of Wisconsin-La Crosse, La Crosse, WI, United States; ^3^Department of Neuroscience, Imaging and Clinical Sciences, University “G. d’Annunzio” of Chieti-Pescara, Chieti, Italy; ^4^Behavioral Imaging and Neural Dynamics Center, Department of Medicine and Aging Sciences, University “G. d’Annunzio” of Chieti-Pescara, Chieti, Italy; ^5^IMT School for Advanced Studies Lucca, Lucca, Italy; ^6^Independent Sport Psychology Consultant, Rome, Italy

**Keywords:** sport psychology, adversity, isolation, psychological well-being, performance, coronavirus

## Abstract

The recent global outspread of the COVID-19 pandemic has influenced the lives of people across multiple countries including athletes, coaches, and supporting staff. Along with everybody else, coaches found themselves constrained to an at-home self-isolation, which limited their ability to normally engage with their profession and to interact with their athletes. This situation may also have impacted their own psychological well-being. With this study, we explored coaches’ perceptions of stress in relation to their emotion regulation strategies depending upon their gender and competitive level (elite vs. non-elite). A sample of 2272 Italian coaches were surveyed during the period of lockdown. Mean values for perceived stress and emotion regulation strategies were compared to normative data of the two instruments as reported in the original studies. Furthermore, two Multivariate analyses of variance (MANOVAs) were completed to observe the potential differences in the coaches’ emotion regulation strategies and perception of stress. Finally, a blockwise regression analysis was run to assess how coaches’ emotion regulation strategies impacted upon their perception of stress. Both women and men reported higher levels of perceived stress than those reported in the normative data. Similarly, average scores for emotion regulation strategies were significantly different from those reported for normative data, in particular, coaches reported slightly higher use of emotion regulation strategies than participants in the original study. Significant gender-based differences emerged in terms of emotional regulations, with men adopting more suppression than women. No differences by competitive level were found. In terms of perceived stress, male coaches and elite coaches showed to be more in control of the situation (positive stress) than female coaches and non-elite coaches, respectively, while women experienced more negative stress than men. The blockwise regression evidenced how reappraisal resulted to be predictive in helping coaches to reduce their perception of stress, while suppression predicted higher stress perceptions.

## Introduction

In the last months of 2019 and in the early months of 2020, the surge of the new severe acute respiratory syndrome coronavirus 2 (SARS-CoV-2) affected the whole globe. Between February and March 2020, the outbreak of the Corona Virus Disease (COVID-19) affected most European countries, as well as countries in Asia, Oceania, and North, Central and South America (WHO, 2020). The spread of the virus led the World Health Organization to declare the COVID-19 a “Global Pandemic” (WHO, 2020). In this scenario, Italy represented a peculiar case characterized by a rapid escalation much earlier in the global pandemic than almost any other country.

In late February 2020 the Italian government declared a mandatory home isolation, initially for specific regional areas, which was then extended to the entire national territory on the 9th of March (Gazzetta Ufficiale, 2020). This mandatory period of national lockdown (56 days of home isolation) limited any possibility of outdoor and in person social activities, with exceptions for essential workers, or for health-related and other essential reasons (e.g., weekly grocery). In the present article, we used the expression “COVID-19 lockdown” to refer to this situation, as this led to the development of chronic stressors, with negative effects for mental and physical well-being (Cao et al., 2020; Flesia et al., 2020; Fuzéki et al., 2020).

The COVID-19 global pandemic also impacted the world of sports and athletics. For example, major national and international sporting competitions were suspended or postponed for 1 year (Corsini et al., 2020; Gallego et al., 2020; Schinke et al., 2020). In addition to its large-scale impact on competition, the COVID-19 lockdown also impacted athletes, coaches, and supporting staff, who were kept away from their usual working (and social) environments (Costa et al., 2020; Di Fronso et al., 2020; Jukic et al., 2020). In addition to limiting their ability to compete in the current season and to prepare for the next, these restrained living conditions and consequential forced physical isolation, may also have negatively impacted the psychological wellbeing of athletes, coaches and supporting staff. In this study, we are particularly interested in coaches’ perception of stress and their ability to regulate their emotions during this once-in-a-lifetime situation.

From a theoretical standpoint, perceived stress has been operationalized as a twofold construct (Cohen et al., 1983). On one hand, positive stress is conceptualized as the perception of being in control of a situation, while, on the other hand, negative stress is conceptualized as the perception of lacking control over a situation and the consequential sense of cognitive overwhelmingness (Cohen et al., 1983; Cohen and Williamson, 1988). High levels of perceived stress can be considered as a lack of positive stress and an abundance of negative stress (Cohen et al., 1983). The perception of stress has been widely investigated among coaches in non-emergency situations, often linking higher perceptions of stress to burnout symptoms (e.g., Kelley et al., 1999; Malinauskas et al., 2010; Knight et al., 2013). Collectively, these findings seem to suggest that women perceive high levels of stress more commonly than men. However, this body of work presents some mixed results about the possible differences between coaches working at different levels of competition. While some scholars did not find any differences in stress perceptions (Kelley et al., 1999), others reported how expert coaches seem to be more prone to burnout than non-expert coaches (Malinauskas et al., 2010).

The relationship between perceived stress and emotion regulation has been explored also in healthcare professionals, as well as in clinical and non-clinical populations (e.g., Moore et al., 2008; Extremera and Rey, 2015; Katana et al., 2019). While some scholars explored the potential predictive role of emotion regulation on stress perceptions (Katana et al., 2019), others explored the moderating effect that emotion regulation may have on the relationship between stress perception and well-being (Extremera and Rey, 2015). In particular, Katana et al. (2019) found that the reappraisal of positive emotions was associated with lower levels of perceived stress, while the suppression of positive emotions was associated with greater levels of perceived stress. When investigating the moderator role of emotion regulation on the relationship between perceived stress and well-being, Extremera and Rey (2015) found that the ability to regulate one’s own emotions can minimize the negative impact of perceived stress on an individual’s well-being. To our knowledge, no studies have explored the relationship between emotion regulation strategies and perceived stress in a sample of sports coaches.

The model of emotion regulation proposed by Gross (1998, 2001) posits that, while experiencing an emotion-generation process, an individual can adopt two main and consequential regulatory strategies: one focused on the antecedent, “reappraisal,” and one focused on the emotional response, “suppression.” Reappraisal consists in changing the way one thinks about a situation and it is generally implemented early on in the process. Suppression involves inhibiting the expressions of emotions and it is generally implemented later on in the process. For example, one could experience sadness for a certain situation, but can be able to change his perspective and then experiencing relief. If this reappraisal strategy does not work, the person can still try to suppress their sadness, but, while this may lead to lesser negative emotions, it may also reduce positive emotions. Gross (1998; 2001) also described how the way an individual reappraises a specific situation influences the way they express emotions. Gross and John (2003) highlighted how reappraisal strategies lead to greater positive emotions, interpersonal functioning and well-being and lesser negative emotions. Suppression strategies – generally more commonly implemented by men – are less adaptive than reappraising strategies and may lead to poorer interpersonal functioning and wellbeing (Gross and John, 2003). Scholars have shown how the same emotion regulation model can explain the reaction of coaches and athletes in sporting contexts (e.g., Lane et al., 2012; Davis and Davis, 2016).

To the best of our knowledge, no published studies have explored the potential impact that a forced isolation experience, like the one during the lockdown implemented in the efforts against COVID-19, may have on coaches’ stress and on their ability to manage it through emotional regulation. In the present study, we aimed to explore how the prolonged experience of imposed lockdown impacted coaches’ perception of stress and their emotion regulation strategies. Moreover, we wanted to explore possible existing differences based on coaches’ reported gender identities as well as their level of competition (elite vs. non-elite). Finally, we aimed to explore the predictive role of emotion regulation strategies toward perceived stress. Despite the explorative nature of the present study, we hypothesized that during the lockdown: (a) coaches perceive higher levels of stress and implement more emotion regulation strategies when compared to the normative data derived from the original developmental studies; (b) elite coaches would perceive higher levels of stress when compared to non-elite coaches; (c) women would experience higher levels of perceived stress than men; (d) men would implement more suppression emotion regulation strategies than women; and (e) emotion regulation strategies would impact on the coaches perceived level of stress, with reappraisal predicting lower levels of perceived stress and suppression predicting higher levels of perceived stress.

## Materials and Methods

### Sample

A sample of 2272 Italian coaches was surveyed between April and early May 2020, period during which they were confined at home due to COVID-19 related forced lockdown. Coaches, predominantly men (*n* = 1,532 men; *n* = 740 women), reported an age between 18 and 80 years (*M*_*age*_ = 41.57; *SD_age_* = 11.99). Participants reported to coach individual (e.g., skating, swimming, gymnastics, dancesport, fencing, and tennis) and team (e.g., rugby, soccer, volleyball, and basketball) sports at different competitive levels (i.e., local, regional, national, and international). Following the classification used by Swann et al. (2015) for athletes, coaches were classified as “elite” (*n* = 889), when working at the national and international levels, and “non-elite” (*n* = 1,383), when working at the local and regional levels.

### Measures

The participants were asked to complete an online survey composed by a battery of questionnaires, encompassing: (a) a demographic information form; (b) the Perceived Stress Scale (PSS; Cohen and Williamson, 1988); and (c) the Emotion Regulation Questionnaire (ERQ; Gross and John, 2003).

#### Demographic Information

After completing the informed consent, each participant was asked to respond to four demographic questions. Specifically, they were asked about their gender, age, and they were also asked to report the typology of sport they were coaching at the time of the survey (individual or team sport) and their competitive level (local, regional, national, or international).

#### Perceived Stress Scale

The Italian version of the Perceived Stress Scale (IPSS-10 – Mondo et al., 2019) consists of 10 items divided in two sub-dimensions, respectively, labeled as positive and negative perceived stress. Items were scored on a 5-point Likert scale ranging from 0 (never) to 4 (very often). The IPSS items are designed to evaluate whether respondents find their lives unpredictable, uncontrollable, and overloading (Cohen et al., 1983; Mondo et al., 2019). All items were introduced by the stem “In the last month…,” with four items measuring “positive stress” and the remaining six items measuring “negative stress.” While the two dimensions can provide independent scores, they can also contribute to the computation of a total score. The total score per each individual was calculated as the sum of all items scores, after reversing the positive stress items scores. Reliability values for Cronbach’s alphas in the Italian version (Mondo et al., 2019) are all from acceptable to good and range from 0.74 for the aggregate score, 0.72 for the “positive stress” subscale and 0.84 for the “negative stress subscale.

#### Emotion Regulation Questionnaire

The Italian version of the ERQ (Balzarotti et al., 2010) consisted of two dimensions and 10 items scored using a 7-point Likert scale ranging from 1 (strongly disagree) to 7 (strongly agree). The first dimension, labeled “reappraisal” consisted of six items while the second dimension, “suppression,” consisted in the remaining four items. The Italian version of the instrument demonstrated acceptable psychometric properties with Cronbach’s alpha values of 0.84 for the “reappraisal” subscale and 0.72 for the “suppression” subscale (Balzarotti et al., 2010).

### Procedure

Coaches were mainly recruited using convenience and snowball sampling techniques. The online survey was posted on the websites and social networks of individual national sport governmental bodies. Moreover, the survey distribution was also supported by the School of Sport of the Italian Olympic Committee (SDS-CONI) by posting the survey link on their main page generally visited by athletes and coaches from the entire national territory. Concurrently, the researchers engaged in a snowball sampling process, by directly inviting coaches in their own personal and professional networks to participate in the study and by asking them to forward the invitation to their colleagues (Sadler et al., 2010). Once the coaches accessed the online survey, they were provided with a brief description of the study and were asked to confirm their agreement to participate in the study as described in the informed consent. The study was conducted in accordance with the declaration of Helsinki and received approval by the institutional review board from the last author’s university.

### Data Analysis

At first, we completed an initial exploratory data analysis. Screening the sample, we removed 139 cases (i.e., lack of information about level of competitiveness or typology of sport, repeated or incomplete cases, or response patterns). As indicated in the informed consent, participants could exit the survey at any point, however, if interested in taking part in the study, they were required to complete it in full. For this reason, no missing values were detected exploring the dataset. Data was then analyzed using IBM SPSS 20.0 for observing distribution and reliability.

We first compared the mean scores (and standard deviations) of coaches’ perceptions of stress and emotion regulation strategies – suppression and reappraisal – reported during the COVID-19 lockdown with those reported by the authors of the two questionnaires in the original developmental studies (Cohen and Williamson, 1988; Gross and John, 2003). This decision was made based on the similarities in size and demographic characteristics between the samples in the three studies. Moreover, Cohen and Williamson (1988) recommended the use of the values of the means and standard deviations from their samples as “norms for use in evaluating scores from other samples” (p. 61). To provide a detailed overview of the extent to which the COVID-19 lockdown impacted on the perception of stress and emotion regulation, we compared the data of women and men separately through calculation of Cohen’s *d* (Cohen, 1988). For Cohen’s *d*, effect sizes of 0.20, 0.50, and 0.80 are considered small, medium, and large effects, respectively. We then completed two Multivariate Analyses of Variance (MANOVAs) aiming to explore the possible differences in terms of perceived stress and emotion regulation strategies between men and women as well as elite coaches and non-elite coaches. Effect sizes were calculated using partial eta square, η_*p*_^2^ (Lakens, 2013), with 0.01, 0.06, and 0.14 considered small, medium, and large effects, respectively (Cohen, 1988). Finally, a blockwise regression was run to observe the predictive role of emotion regulation strategies toward perceived stress. In line with Gross and John’s (2003) theory, the emotion reappraisal variable was introduced first in the blockwise regression model, followed by emotion suppression variable.

## Results

An examination of histograms, and values of skewness and kurtosis showed adequate levels of normality in the collected data leading to the decision that further parametric tests could be undertaken. Internal consistency reliability in our sample was acceptable to good for both the PSS and the ERQ. In particular, for the PSS, Cronbach’s alpha values were 0.74 for the “positive stress” subscale, 0.79 for the “negative stress” sub-scale, and 0.82 for the aggregated score, while for the ERQ, they were 0.82 and 0.73 for the “reappraisal” and “suppression” sub-scales, respectively.

The comparison between data related to the perception of stress collected during COVID-19 lockdown and the normative data (see Table 1) showed significant differences (and medium effect sizes), with increased perceived stress during lockdown for both men (difference = 2.840, *t* = 13.513, df = 2936, *p* < 0.0001, Cohen’s *d* = 0.49) and women (difference = 4.310, *t* = 14.006, df = 1664, *p* < 0.0001, Cohen’s *d* = 0.69). Despite the small effect sizes observed, also suppression and reappraisal mean scores were significantly higher during COVID-19 lockdown if compared with normative data (see Table 1), for both men (suppression: difference = 0.320, *t* = 5.389, df = 2077, *p* < 0.0001, Cohen’s *d* = 0.27; reappraisal: difference = 0.420, *t* = 8.630, df = 2077, *p* < 0.0001, Cohen’s *d* = 0.43) and women (suppression: difference = 0.350, *t* = 5.808, df = 1674, *p* < 0.0001, Cohen’s *d* = 0.28; reappraisal: difference = 0.422, *t* = 8.371, df = 1674, *p* < 0.0001, Cohen’s *d* = 0.41).

**TABLE 1:** Means (and standard deviations) of Italian coaches’ perceived stress, suppression, and reappraisal prior to and during COVID-19 lockdown.

	Normative data		During COVID-19	
	Women	Men	Women	Men
Perceived stress	13.70 (6.60)	12.10 (5.90)	18.01 (5.76)	14.94 (5.49)
Suppression	3.14 (1.18)	3.64 (1.11)	3.49 (1.28)	3.96 (1.22)
Reappraisal	4.61 (1.02)	4.60 (0.94)	5.03 (1.02)	5.03 (0.99)

*Perceived stress–Original sample: Women = 926; Men = 1,406; During COVID-19 sample: Women = 740; Men = 1,532; Suppression and reappraisal–Original sample: Women = 936; Men = 547; During COVID-19 sample: Women = 740; Men = 1,532.*

Examining the relationship between participants’ gender and competitive level, we completed two MANOVAs. In the first MANOVA, we examined a 2 × 2 matrix with gender and competitive level as fixed factors and the two sub-dimensions of the PSS as dependent variables. Results showed significant differences both for gender [Wilks’ λ = 0.938, *F*(2,2267) = 75.35, *p* < 0.00, η_*p*_^2^ = 0.062, observed power >0.999] and competitive level [Wilks’ λ = 0.995, *F*(2,2267) = 5.61, *p* < 0.00, η_*p*_^2^ = 0.005, observed power = 0.859]. In particular, women exhibited lower “positive stress,” *F*(1,2271) = 76.03, *p* < 0.00, η_*p*_^2^ = 0.032, observed power >0.999, and higher “negative stress,” *F*(1,2271) = 134.9, *p* < 0.00, η_*p*_^2^ = 0.056, observed power >0.999. Non-elite coaches also reported lower “positive stress” levels, *F*(1,2271) = 10.27, *p* < 0.00, η_*p*_^2^ = 0.005, observed power = 0.893, when compared with elite coaches.

In the second MANOVA, we examined a 2 × 2 matrix (gender × competitive level) with the dimension of the ERQ as dependent variables. Significant differences for gender emerged [Wilks’ λ = 0.969, *F*(2,2267) = 75.35, *p* < 0.00, η_*p*_^2^ = 0.031, observed power >0.999], with men more prone to use “suppression” as an emotion regulation strategy, *F*(1,2271) = 70.31, *p* < 0.00, η_*p*_^2^ = 0.030, observed power >0.999. No other significant differences emerged from the analysis. Means and standard deviations for women and men, and for elite and non-elite coaches on the PSS and the ERQ are shown in Table 2.

**TABLE 2:** Means and Standard Deviations for the PSS and the ERQ.

		Perceived Stress Scale		Emotion Regulation Questionnaire	
		Positive stress	Negative stress	Reappraisal	Suppression
Gender	Women	9.66^a^ (2.64)	11.67^a^ (4.02)	5.03 (1.02)	3.49^a^ (1.29)
	Men	10.66^a^ (2.28)	9.60^a^ (3.68)	5.03 (0.99)	3.96^a^ (1.22)
Competitiveness	Elite	10.49^b^ (2.45)	10.30 (3.92)	5.05 (1.01)	3.84 (1.28)
	Non-elite	10.23^b^ (2.45)	10.25 (3.91)	5.02 (1.00)	3.79 (1.25)

*^*a*^Significantly different for men/women. ^*b*^Significantly different for elite/non-elite coaches.*

Finally, we conducted a blockwise regression analysis to observe the impact of emotion regulation strategies on the perception of stress. Results of the regression [*R*^2^ = 0.068; *F*(2,2271); *p* < 0.001] showed how both reappraisal and suppression regulations were predictive of perceived stress, although the model was only able to explain 7% of the variance. Specifically, reappraisal was a negative predictor (β = −0.239; *p* < 0.001), while suppression was a positive predictor (β = 0.137; *p* < 0.001) of perceived stress. Which means that individuals with high capacity of reappraisal have a protective factor toward the perception of stress. On the other hand, participants that suppressed their cognitive emotion were prone to experience more stress (see Table 3).

**TABLE 3:** Predictors of perceived stress.

	B	SEB	β	*p*	*C.I.*
Reappraisal	−1.308	0.112	−0.239	0.000	−1.528; −1.089
Suppression	0.597	0.089	0.137	0.000	0.423; 0.771

## Discussion

In the present study, we explored the levels of perceived stress experienced by coaches during the period of lockdown due to the COVID-19 emergency. Two months of lockdown may represent a highly stressful experience for coaches, and distressed coaches can exhibit emotions and engage in behaviors which may be detrimental for athletes’ mental well-being (Davis and Davis, 2016). Thus, it was important to study coaches’ emotional reactions to the compulsory lockdown in response to the COVID-19 emergency. With this study, we contributed to extend the current body of knowledge focused on perceived stress and emotion regulation among coaches during periods of mandatory or forced isolation.

Overall, findings may suggest that the COVID-19 lockdown had a harmful effect on coaches’ perceived stress, which was significantly higher than the levels of stress reported in the normative data. The observed detrimental impact on both men and women might be due to the uncommon characteristics of the COVID-19 crisis. Moreover, the postponement and/or cancelation of all competitions and sport-related events due to this emergency may also have contributed to worsen the perceived stress of athletes (e.g., Di Fronso et al., 2020) as well as coaches. As a consequence, during the COVID-19 confinement, male and female coaches reported levels of emotion regulation strategies greater than those reported in the normative data.

In the uniqueness of this COVID-19 emergency, female coaches reported to experience higher level of perceived stress than male coaches. These results are in line with past literature (Kelley and Gill, 1993; Kelley, 1994; Kelley et al., 1999), but might have been exacerbated over the period of the forced isolation due to the mandatory lockdown. In particular, three aspects might have played a role in causing higher levels of perceived stress among women: (a) pre-existing gender differences; (b) perception and experienced social support; and (c) organizational support. First, the fact that gender differences exist in perceived stress has been previously established (e.g., Kelley and Gill, 1993) and it emerged, in this period of lockdown, also among Italian athletes, with women experiencing more stress than men (Di Fronso et al., 2020). With regards to social support, scholars previously showed how male coaches who experienced social support also reported reduced levels of perceived stress (Kelley, 1994). The effect of social support on perceived stress may also have been experienced by the coaches participating in the present investigation with men more prone to seek online social interactions than women, thus potentially receiving higher levels of social support. Finally, at an organizational level, female coaches may have felt less supported than male coaches. Previous literature shows how within sporting organizations, women generally receive lower salaries and experience lesser job stability than men (Carson et al., 2018). These gender-based organizational differences, may have been exacerbated during the COVID-19 lockdown, leading women coaches to experience lower levels of job security and financial stability.

In terms of differences by competitive level, elite coaches reported to be more in control of the situation (“positive stress”) and these results did not support our hypothesis. In fact, we expected to find elite coaches to be more stressed than non-elite coaches as they may have greater job responsibilities and more professional expectations for sporting results. However, elite coaches may also work in highly structured sport organizations able to provide them with higher level of support to their remote professional activities (Larner et al., 2017; Wagstaff et al., 2018; Arnold et al., 2019). Moreover, these coaches may have worked with athletes who were able to continue their training also during lockdown, due to the organizational instrumental support (Fletcher and Arnold, 2017). On the other hand, instead, non-elite coaches may have been working with athletes who divested from their athletic role (Costa et al., 2020). Finally, due to their highly competitive working conditions and environment, elite coaches may have developed the ability to effectively and positively cope with high stressful situations (Mellalieu et al., 2009; Fletcher and Scott, 2010; Olusoga et al., 2012).

Findings from the present investigation are somehow in contrast with previous studies. For example, Kelley et al. (1999) did not find any significant differences in tennis coaches’ perceptions of stress based on their competitive level. Moreover, Malinauskas et al. (2010) reported how experienced coaches were more prone to burnout than less experienced coaches. Several reasons may explain these different results. On one hand, Kelley and colleagues explored a specific sample of tennis coaches and their study was not performed under a pandemic situation. On the other hand, Malinauskas et al. (2010) studied experienced coaches, who are not necessarily elite coaches, and focused on their experience of burnout, which, although related to perceived stress, is a different construct. In light of Malinauskas et al. (2010) results, it may be interesting to extend the present investigation and explore more in detail the potential differences in the levels of perceived stress and emotional regulations between coaches in relation to both their level of experience and presence of burnout symptoms.

Gender-based differences in emotion regulation and, specifically with regards to “suppression” regulation, confirm the existing literature showing that men tend to suppress their emotions more commonly than women (e.g., Gross and John, 2003). From our study, it also appears that emotion regulation strategies played a role in predicting the perceived stress of coaches during the COVID-19 lockdown. In particular, although with a small effect, we observed how the ability of Italian coaches to reappraise the situation derived from the COVID-19 emergency helped them to reduce their experience of stress. On the contrary, the use of suppression as a strategy predicted an increase of perceived stress levels among the coaches participating in our study. These results combined seem to suggest that men have a more common tendency to adopt a less adaptive emotion regulation strategy then women. To change this trend, it could be important to teach male coaches to use more reappraisal, instead of suppression, as an emotion regulation strategy.

Based on our results, some practical implications can be provided to coaches, sport psychology practitioners, and organizations on how to intervene to reduce stress, disclose one’s own emotions and reappraise the situation. At individual/group level, coaches could benefit from learning and possibly engaging in some mindfulness meditation activities with the scope of regulating their levels of perceived stress (Austin, 1997; Longshore and Sachs, 2015). Coaches could also engage in written emotional disclosure interventions to promote and support their own emotional expressivity (Lumley and Provenzano, 2003). Sport psychology practitioners could support coaches in these efforts by directly developing and implementing these techniques as well as other interventions to help coaches to learn how to regulate their own emotions during stressful situations (Kivity and Huppert, 2016; Dryman and Heimberg, 2018). Additionally, practitioners could also share instructional material with coaches to engage them in self learning. At an organizational level, sports clubs and federations could build support systems to help manage stressful situations by either providing training or giving access to coaches to sport psychology practitioners (Taylor, 1992). Due to the unique nature of a forced isolation as the one due to the COVID-19 emergency, the possibility to access web-based interventions might be helpful to support coaches, alleviating their stress and potentially protecting them from negative mental health symptoms (Morris et al., 2015).

We also acknowledge some limitations of this study. For example, we had to rely on a convenience sample, which, regardless of its size, lacks randomization, limiting our ability to generalize our findings. Additionally, the results of the comparison to the datasets derived from the original developmental studies for the PSS (Cohen and Williamson, 1988) and the ERQ (Gross and John, 2003) may not have been optimal. For example, these differences may have not been necessarily due to the impact of the mandatory lockdown during the COVID-19 emergency on the participating coaches, and instead could be impacted by the specific characteristics of the surveyed population (i.e., coaches vs. non-coaches). It should also be noted that this study is based on a cross-sectional design and the inferences on how stress perceptions and emotion regulation strategies varied across time are limited to this comparison with normative data. Longitudinal investigations of how the coaches’ levels of perceived stress varied across the whole period of the COVID-19 emergency would allow inferences to be made on the causality and would enrich the literature on this unique situation. Moreover, future studies conducted during the COVID-19 pandemic or similar emergencies leading to forced isolation, could also explore other aspects, such as the level of experience of the coaches and burnout symptoms. Finally, it will be important to take into consideration also the effect that coaches’ emotions have on athletes’ reactions (e.g., Thelwell et al., 2008; Friesen et al., 2018). Since athletes also suffered the effects of the COVID-19 emergency (e.g., Di Fronso et al., 2020), future studies might want to explore the coach-athlete relationship and its association with coaches’ mental health during similar period of high non-sport related stress.

## Conclusion

In conclusion, by exploring the levels of perceived stress and the emotion regulation strategies within a large sample of Italian sports coaches during the period of the COVID-19 lockdown, we found a series of noteworthy results. First, we found that these Italian coaches during the mandatory lockdown reported to experience higher levels of perceived stress and to implement more emotion regulation strategies than those proposed in the normative data. Elite coaches seem to perceive themselves to be more in control of the situation (“positive stress”) than their non-elite counterparts. While female coaches appear to experience higher levels of perceived stress than male coaches, male coaches seem to adopt “suppression” more often as a regulatory strategy than their female counterparts. Lastly, we have observed a small impact of emotion regulation strategies on the coaches’ levels of perceived stress, with reappraisal regulatory strategy predicting lower levels of perceived stress and suppression regulatory strategy predicting higher levels of perceived stress. While we do recognize the peculiarity of this study in this particular period, our findings can be generalized to other cases of forced isolation, such as those due by illness or injury. Indeed, even in those cases, coaches can find themselves confined at home and they can find support in web-based interventions (e.g., Morris et al., 2015; Lane et al., 2016).

## Data Availability Statement

The raw data supporting the conclusions of this article will be made available by the authors, without undue reservation.

## Ethics Statement

Ethical review and approval was not required for the study on human participants in accordance with the local legislation and institutional requirements. The patients/participants provided their written informed consent to participate in this study.

## Author Contributions

All authors listed have made a substantial, direct and intellectual contribution to the work, and approved it for publication.

## Conflict of Interest

The authors declare that the research was conducted in the absence of any commercial or financial relationships that could be construed as a potential conflict of interest.
